# Secondary Traumatic Stress in Italian Police Officers: The Role of Job Demands and Job Resources

**DOI:** 10.3389/fpsyg.2020.01435

**Published:** 2020-06-26

**Authors:** Daniela Acquadro Maran, Margherita Zito, Lara Colombo

**Affiliations:** ^1^Department of Psychology, Università di Torino, Turin, Italy; ^2^Department of Business, Law, Economics and Consumer Behaviour “Carlo A. Ricciardi”, Università IULM, Milan, Italy

**Keywords:** secondary traumatic stress, police officers, burnout, job demand, job resource

## Abstract

Police officers are among the workers most exposed to acute or chronic stressful events, which compromises their psychosocial well-being and physical health. Exposure to traumatic events, human suffering, problematic situations and episodes of violence can cause psychological damage and lead to the development of secondary traumatic stress. The aim of this research is to explore the effect of job demands and job resources on secondary traumatic stress in police officers. To better understand this phenomenon and its consequences in this population, police officers were compared with health care professionals working as first responders. An *ad hoc* questionnaire was administered to 112 and 286 health care professionals. The findings showed that compared with health care workers, police officers suffer from secondary traumatic stress to a greater extent. Moreover, the results showed that some police officers suffered more than health care professionals regarding certain consequences of secondary traumatic stress, such as negative emotions and burnout. This study suggests implications and offers insights for both police officers and the organizations in which they work: police officer organizations should contribute to preventing the phenomenon of secondary traumatic stress by proposing programs that implement resilience training and adaptive coping strategies.

## Introduction

A literature analysis has shown that police officers (hereinafter POs) are among the workers most exposed to acute or chronic stressful events (VanDercar et al., 1980), which compromises their psychosocial well-being and physical health (Storch and Panzarella, 1996; Kop et al., 1999; Bachynski et al., 2012; Kessler et al., 2014). Kop et al. (1999) affirmed that continued contact with others’ suffering as well as helping or attempting to help persons undergoing traumatic experiences may result in emotional responses. Given the high frequency of their interpersonal relationships with citizens, POs are considered “helping professionals” (e.g., doctors, nurses, psychologists, social workers), who are characterized by their exposure to traumatic events (Maslovaric et al., 2017), human suffering, problematic situations and episodes of violence. This exposure can cause psychological damage and lead to the development of secondary traumatic stress (hereinafter STS; Figley, 1995), which is defined by Baird and Kracen (2006, p. 181) as “a set of psychological symptoms that mimic post-traumatic stress disorder but is acquired through exposure to persons suffering from the effects of trauma.” STS can cause consequences in POs, including disruptions of both their personal and professional identity, anxiety, depression, sleep disorders, intrusive thoughts, maladaptive coping strategies, and negative feelings, such as anger (Saakvitne et al., 1996; Leonard and Alison, 1999; Vrklevski and Franklin, 2008). Moreover, STS can have effects on resilience among POs (MacEachern et al., 2011) and can inhibit the release of negative feelings related to their responsibilities; additionally, STS may impact the experience of fairness and emotive withdrawal from their affective and social environment (Pearlman and Saakvitne, 1995).

As described by MacEachern et al. (2019), other definitions have also been used to describe the consequences that result from working with traumatic events. These definitions include vicarious trauma and compassion fatigue. While vicarious trauma emphasizes cognitive schema changes while still allowing symptomatic stress (McCann and Pearlman, 1990), compassion fatigue is, according to Figley (1995), an effect of secondary traumatic stress, i.e., “compassion is a feeling of deep sympathy or sorrow for another who is stricken by suffering or misfortune, accompanied by a strong desire to alleviate the pain or remove its cause” (p. 7). For this study, the definition of STS by Baird and Kracen (2006) has been used.

### STS and the Job Demands-Resources Model

Violanti and Paton (1999) described stress-related trauma as a product of the interaction among environment, situation, person, support mechanisms and interventions. Considering the theoretical framework of the *job demands-resources model* (JD-R model, Demerouti et al., 2001; Bakker and Demerouti, 2007, 2014), stressors among POs are related to occupational (such as traffic control, patrol activities, crime prevention, criminal investigations, and community services) and job demands (such as, a lack of a support system provided by supervisors and/or colleagues, autonomy in the management of one’s own job, and cognitive control over the work done) (Leino T. et al., 2011; Suresh et al., 2013). Moreover, Burke et al. (2007) underlined that workers with more working experience report lower stress levels than colleagues with fewer years of experience; this outcome is apparently due to their adoption of more effective coping strategies to deal with traumatic events over time (White et al., 1985), having greater autonomy and having more control over the work done. Leino T. et al. (2011) have suggested that the experience of emotional trauma is reduced by the possibility of attaining a higher position within an organization, i.e., one that permits changes in job demands. A better position could also mitigate the cognitive and emotive dissonance in POs that results from controlling citizenship safety and dealing with events that are beyond their control (Violanti, 2006), i.e., the more uncontrollable an event is perceived to be, the higher the risk of developing STS (Carlier et al., 2000). Another explanation for the decrease in stress includes a decrease in direct exposure to traumatic events, as with career progression, POs tend to occupy positions of leadership and command.

However, Brown and Campbell (1990) and Kirkcaldy et al. (1998) report that POs with higher positions report higher levels of stress than patrol POs, relating to supervisors’ engagement in both front-line police duties and their greater exposure to organizational and management stressors.

While it could be possible to alter the organizational demands that may positively influence the perception of stress and its consequences, the occupational demands cannot be changed, as these are job characteristics. Some studies show that organizational stressors have a higher impact on PTSD than operational stressors (Maguen et al., 2009). The consequences of organizational demands could have an impact on the development of STS in POs as well as in other workers (Colombo et al., 2019). An investigation conducted by Sharma (2013) showed that hostile relationships between supervisors and workers negatively affect work performance, stress perception and its consequences; these findings were consistent with previous research (Chopko, 2010; Shane, 2010; Yuan et al., 2011) that found that the management of potentially traumatic events leads to a decrease in psychological problems among traumatized POs. The work of POs also contains important resources that could lead to reduced job demands and consequences, thereby facilitating new strategies to cope with STS (Gillet et al., 2013). These resources include feelings of self-efficacy (individual beliefs about his/her capabilities to achieve a particular task successfully; Bandura, 1977; Chu et al., 2019); emotional support from colleagues, supervisors and family (Bakker et al., 2019); and gratitude from citizens (McCanlies et al., 2014). In particular, Mikkelsen and Burke (2004) examined 766 male and female Norwegian POs and found that work-family conflict was related to psychological health, while Zavala and Kurtz (2016) found that POs that received family support were less likely to adopt maladaptive coping strategies, such as alcohol consumption. Thus, both work-family conflict and family support variables must be considered to better understand the role of families in work-related stress (Pattusamy and Jacob, 2017). Thompson et al. (2005) found that in POs, support from supervisors was related to a decrease in stress. McCanlies et al. (2014) found that gratitude from citizens could mitigate the consequences of traumatic events among POs since gratitude increases the ability to develop resources to address distress.

### STS and Burnout

Burnout is a term coined by Freudenberger (1974) and is used to describe emotional exhaustion, depersonalization, the erosion of idealism, and the loss of self-efficacy resulting from prolonged work (Figley, 1989; Pearlman and Saakvitne, 1995; Bakker and Heuven, 2006). In POs, burnout risk factors are prevalent due to the lack of participation in decision making (Perez et al., 2010), poor social support (Violanti, 1993) and the negative job image of citizens (Lim et al., 2000). According to Injeyan et al. (2011), burnout and STS are distinct phenomena; while burnout is related to prolonged work, STS is related to empathy as well as prolonged exposure to suffering and traumatic events (Figley, 2002). However, STS can influence burnout because STS is a source of stress related to emotional exhaustion; i.e., the emotive impact the PO experiences as a result of the intervention in traumatic events can produce symptoms, such as helplessness and depression. According to McCann and Pearlman (1990) and Figley (1995), as a consequence of their work, POs repeatedly encounter traumatic events through detailed descriptions of what citizens and colleagues have experienced, which may result in STS symptoms. Thus, investigating burnout in relation to STS could provide the opportunity to investigate how STS is related, for example, with the propensity to change jobs.

To better understand STS and its determinants in this specific population, a comparison was made of workers who share the same urban context and the need for intervention in traumatic events as POs. Thus, HCPs working as first responders (i.e., ambulance, emergency units) were included in this study. Duffy et al. (2015) examined 117 HCPs working in Irish emergency departments and found that 64% of the participants met the criteria of STS and that most of them were more prone to use maladaptive strategies to cope with stress. As suggested by Montgomery and Maslach (2019), there is a vast literature about burnout, job demands and job resources among HCPs. Because of this focus, interventions to reduce STS and burnout and to promote individual and organizational resources are well-documented (see, for example, Ray et al., 2013). As a consequence, all over the world, there are examples of interventions to mitigate the consequences of stress and to improve well-being in these workers (Ruotsalainen et al., 2008). In Italy, for example, STS and burnout are issues that are explained in the training to become a nurse (Sirigatti et al., 1988); thus, these workers have an opportunity to recognize the symptoms of STS and burnout and ask for help. In POs, few hours were dedicated to psychosocial risk; thus, the STS and its consequences were not analyzed in depth^1^.

### Current Study

The aims of this research are as follows: 1. to explore the effect of job demands (workload, emotive dissonance, citizens’ demands, cognitive load, work-family conflict) and job resources (job autonomy, role clarity, organizational support, family support, resilience, secondary trauma self-efficacy) on STS in POs; 2. to identify differences in STS, job demands, job resources, well-being and malaise at work scores between POs and health care professionals (HCPs) working as first responders. POs are workers who interact with individuals who have experienced one (or more) traumatic event (Cornille and Meyers, 1999; Garbarino et al., 2011). As described by MacEachern et al. (2019), these workers “often must share the emotional burden of the trauma, bearing witness to damaging or cruel circumstances that individuals have experienced, and acknowledge the existence of terrible and traumatic events in the world” (p. 166). The originality of this study lies in the fact that, to the best of our knowledge, this is the first time an investigation on STS has been conducted among Italian POs. The participants consisted of POs working in the municipal police of a large city in northern Italy. In this police force, the POs work within the community, and they perform a range of tasks related to the needs of the community (e.g., interventions in road accidents, eviction notices, domestic violence, stalking) (Delvino, 2008). Police services are provided 24 h a day, 7 days a week. At present, this city is the fourth largest Italian municipality per population and the third most economically productive complex in the country. As described in previous research (Acquadro Maran et al., 2018, p. 2), “this police force was established more than 200 years ago in Italy, and there are 10.4 police officers for every 10,000 inhabitants.”

The hypotheses of this study are as follows: job demands (cognitive load, workload, user demands, emotional dissonance, work-family conflicts) are positively related to STS (h1); job resources (job autonomy, role clarity, supervisor and colleagues support, family support, resilience, secondary trauma self-efficacy) are negatively related to STS (h2); and compared with HCPs, POs suffer from higher levels of STS and malaise at work (h3).

## Materials and Methods

This research conformed to the provisions of the Declaration of Helsinki in 1995, as revised in the Edinburgh meeting of 2000 (World Medical Association, 2001). All the relevant ethical guidelines were followed, including compliance with the requirements of Italian legislation. Since there was no medical treatment or other procedures that could cause biological, psychological, or social harm to the participants involved, additional ethical approval was not required.

### Participants and Procedures

The chief of the municipal police and the directors of health services were informed about the aim of the research and agreed to collaborate. The letter of informed consent given to both the chiefs and the participants clearly described the goals of the research, the voluntary nature of participation, the types of data investigated and their statistical processing, and the anonymity of the procedures employed. No one, including the officers, received any compensation for their participation. The data were collected with the cooperation of the municipal police and health service management, which gave us the opportunity to administer the same questionnaire to both POs and HCPs. The POs were attending a training course about administrative procedures. Researchers administered the questionnaire at the beginning of the lesson. All of the POs who participated in the investigation worked in operational services; i.e., these POs are responsible for enforcing the law, intervening directly in cases of assault (e.g., child maltreatment), investigating crimes and ensuring safety (e.g., on the roads). Regarding the HCPs, the questionnaire was administered at the end of their work shift. All of the HCPs that participated in the investigation worked in the emergency department as first responders (e.g., traffic accidents).

### Measure

For this investigation, an *ad hoc* questionnaire was administered with the following measures:

#### Secondary Traumatic Stress (General Index)

The Secondary Trauma Stress Scale by Bride et al. (2004) was used to assess STS. This scale consists of 17 items scored on a 5-point scale, ranging from 1 (never) to 5 (very often). The reference time period for the items is “last week.” An example item is “Reminders of my work with citizens upset me.” The scale comprises three subscales: *intrusion* (5 items; alpha in this study 0.77; alpha in original study 0.80), which refers to thoughts and images of others’ trauma; *avoidance* (7 items; alpha 0.77; alpha in original study 0.87), which refers to emotional and situational fatigue with others’ pain; and *arousal* (5 items; alpha 0.76; alpha in original study 0.83), which is linked to negative emotions and unpleasant conditions. Scores for the STS general index are obtained by summing all items. Cronbach’s alpha for the whole scale in this study was 0.89, and the Cronbach’s alpha in the original study was 0.93.

#### Well-Being and Malaise at Work

##### Positive and negative emotions at work

Warr’s scale (Warr, 1990) was used. This scale consists of six items and is scored on a Likert scale from 1 (never) to 6 (all of the time). The reference time period for the items is “last month.” The participants were asked, when thinking of the preceding few weeks, how much of the time their job had made them feel, e.g., happiness or sadness. Cronbach’s alpha was 0.85 for the positive emotions scale (6 items) and 0.81 (6 items) for the negative emotions scale.

##### Work satisfaction

The COPSOQ II by Pejtersen et al. (2010) was used to measure work satisfaction, i.e., the perception that work enables the satisfaction of important personal values connected with work. This scale consists of 6 items scored from 1 (very unsatisfied) to 5 (very satisfied). An example item is “Regarding your work in general, how pleased are you with your work prospects?” Cronbach’s alpha in this study was 0.83, and in the original study, it was 0.82.

##### Burnout (general index)

The Maslach Burnout Inventory – General Survey (MBI-GS) by Schaufeli et al. (1996) was used to assess *exhaustion* (5 items, alpha 0.87; e.g., “I feel burned out from my work”), cynicism (5 items, alpha 0.78; e.g., “I have become less enthusiastic about my work”), and professional efficacy (6 items, alpha 0.74; e.g., “I feel I am making an effective contribution to what this organization does”). The 16 items are scored as follows: 0 (never), 1 (a few times a year or less), 2 (once a month or less), 3 (a few times a month), 4 (once a week), 5 (a few times a week), and 6 (every day). Scores for the *burnout* general index are obtained by summing all items. Cronbach’s alpha was 0.72. Bakker et al. (2002), in their general survey across occupations, found Cronbach’s alpha coefficients ranging from 0.84 to 0.90 for exhaustion, 0.74 to 0.84 for cynicism, and from 0.70 to 0.78 for professional efficacy.

#### Job Demands

##### Workload

The Job Content Questionnaire (JCQ) by Karasek (1985) and Karasek et al. (1998) was used to measure the content of the respondent’s job. The scoring ranges from 1 (strongly disagree) to 6 (strongly agree). This scale consists of seven items; an example is “My job requires working very fast.” Cronbach’s alpha in this study was 0.72.

##### Cognitive load

The 4-item scale by Bakker et al. (2003) was used to measure the cognitive load. The scoring ranges from 1 (never) to 5 (always). An example is “Your job requires a lot of concentration.” Cronbach’s alpha in this study was 0.81.

##### Citizens’ demands

Dormann and Zapf (2004) customer-related social stressors (Italian adaptation; Taddei and Vanni, 2008) scale was used to assess the requests made by citizens to address their traumatic events. The response scale ranges from 1 (not true at all) to 5 (completely true). An example of one of the 11 items is “Our citizens are unable to wait.” Cronbach’s alpha in this study was 0.92.

##### Emotive dissonance

The 4-item scale by Zapf et al. (1999) was used to measure emotive dissonance. The score ranges from 1 (never) to 6 (always). An example of an item is “Thinking about your work and about your relationship with citizens, how many times have you suppressed your emotions?” Cronbach’s alpha in this study was 0.76.

##### Work-family conflict

Work-family conflict was measured using five items with a 7-point Likert scale from 1 (never) to 7 (always). The items were adapted from the Italian version (Colombo and Ghislieri, 2008) of the Netemeyer et al. (1996) questionnaire. An example item is “The demands of my work interfere with my home and family life.” Cronbach’s alpha in this study was 0.89; Cronbach’s alpha in the adapted Italian version was 0.86. Work-family conflict has been considered a job demand in previous studies (see, e.g., Ghislieri et al., 2017).

#### Job Resources

##### Job autonomy

Job resources items developed by Bakker et al. (2003) were used to measure autonomy and control at work. The three items of the scale are measured on a five-point scale from 1 (never) to 5 (often). An example item is “I am able to decide myself how to execute my work.” Cronbach’s alpha in this study was 0.88.

##### Organizational support

An 8-item scale by Caplan et al. (1975) was used to measure support from supervisors and colleagues. The scores range from 1 (very rarely) to 6 (often). An example item is “How much does [supervisor or colleague] help you to get your job done.” Cronbach’s alpha was 0.94 for Supervisors’ support scale (4 items) and 0.70 for Colleagues’ scale (4 items).

##### Role clarity

*Ad hoc* scales were constructed to measure role clarity (6 items). The scores range from 1 (strongly disagree) to 6 (strongly agree). An example item is “How clear are you about the goals and objectives for your team?” Cronbach’s alpha in this study was 0.82.

##### Family support

This scale, developed by King et al. (1995), was used to measure emotional family support. The items are measured on a 6-point scale from 1 (never) to 6 (often). An example of one of the 15 items is “When something at work is bothering me, members of my family show that they understand how I’m feeling.” Cronbach’s alpha in this study was 0.82.

##### Secondary trauma self-efficacy (STSE)

This scale, developed by Cieslak et al. (2013), was used to assess the perceived ability to cope both with the demands from work with traumatized citizens and the ability to deal with STS symptoms (12 items). The score ranges from 1 (very incapable) to 7 (very capable). An example item is “How capable am I of controlling recurring distressing thoughts or images about these people?” Cronbach’s alpha in this study was 0.89, and Cronbach’s alpha in the original study was 0.87.

##### Resilience

This scale, developed by Di Fabio and Palazzeschi (2012), was used to measure resilience. The items are measured on a five-point scale from 1 (never) to 5 (often). An example of one of the 10 items is “I think I’m a strong person.” Cronbach’s alpha in this study was 0.82, and Cronbach’s alpha in the original study was 0.85.

### Data Analysis

Data analysis was performed using IBM SPSS 25 (Statistical Package for Social Science) and involved descriptive statistics (means, standard deviations) and alpha reliabilities (α – Cronbach’s alpha) for each scale, correlations (Pearson’s r) between the variables, and *t*-tests for independent samples to understand the differences between POs and HCPs in the perception of their STS, job demands, job resources, well-being, and malaise outcomes; the analysis also included hierarchical multiple regression analysis (method: enter), which included STS as the dependent variable.

## Results

An analysis of the demographic characteristics of the participants shows that the sample was composed of 112 POs and 286 HCPs. Among the POs, 27.7% were females, and 71.4% were male. The mean age of the participants was 46.12 years (*SD* = 8.3); 22.3% were single (or never married at the time of the study), 65.2% were married or cohabitating, 8.9% were separated/divorced/widowed, and 67.9% had children. The mean job tenure of the participants was 17.40 years (*SD* = 7.98). Among the HCPs, 44.8% were females, and 54.5% were male. Their mean age was 38.65 years (*SD* = 12.56); 46.2% were single, 40.2% were married or cohabitating, 13.3% were separated/divorced/widowed, and 43.7% had children. Their mean job tenure was 11.60 years (*SD* = 9.68).

Regarding descriptive statistics (Table 1), in general, in both groups of employees, the means of STS are not very high (POs: *M* = 2.17, *SD* = 0.63; HCPs: *M* = 1.62, *SD* = 0.52), whereas the means value of job demands, in particular workload (POs: *M* = 4.16, *SD* = 0.95; HCPs: *M* = 3.65, *SD* = 0.86), emotive dissonance (POs: *M* = 3.96, *SD* = 1.00; HCPs: *M* = 3.90, *SD* = 0.85), citizens’ demands (POs: *M* = 4.88, *SD* = 0.89; HCPs: *M* = 4.73, *SD* = 0.90), cognitive load (POs: *M* = 4.24, *SD* = 0.69; HCPs: *M* = 4.27, *SD* = 0.62), are high. Among job resources, we highlight the high means for resilience (POs: *M* = 3.76, *SD* = 0.53; HCPs: *M* = 3.96, *SD* = 0.59), and STSE (POs: *M* = 5.05, *SD* = 0.99; HCPs: *M* = 5.04, *SD* = 1.03). POs present higher means of STS and job demands, and lower means of job resources than HCPs. This is particularly relevant within supervisors’ and colleagues’ supports, whit a difference specifically higher in the supervisors’ support.

**TABLE 1:** Correlations – POs (under the diagonal) – HCPs (above the diagonal); descriptive analysis of all scales (*M*, *SD*).

	*Pos* M (DS)	*HCPs* M (SD)	1	2	3	4	5	6	7	8	9	10	11	12	13	14	15
1. STS	2.17 (0.63)	1.62 (0.52)	1	0.00	0.03	0.28*	0.09	0.08	0.13*	0.32**	–0.15*	–0.27**	–0.15*	–0.07	0.17**	–0.24**	0.16**
2. Gender (1 = Female)	–	–	–0.08	1	–0.23**	0.12*	–0.00	0.08	0.05	–0.02	–0.06	0.04	–0.06	–0.04	–0.02	–0.03	–0.12*
3. Job tenure	17.4 (7.90)	11.60 (9.67)	–0.08	0.00	1	–0.07	–0.00	0.16*	0.13*	0.04	0.07	0.04	–0.11	0.05	–0.06	0.03	0.05
4. Workload	4.16 (0.95)	3.65 (0.86)	0.14	0.14	0.16	1	0.14*	0.32**	0.45**	0.25**	–0.14*	–0.20**	–0.33**	–0.23**	0.23**	0.03	–0.07
5. Emotive dissonance	3.96 (1.00)	3.90 (0.85)	0.20*	0.05	–0.17	0.24*	1	0.25*	0.13*	0.07	0.00	0.03	0.11	0.03	0.12*	0.11	0.21*
6. Citizens’ demands	4.88 (0.89)	4.73 (0.90)	0.08	0.08	0.03	0.21*	0.27**	1	0.32**	0.23**	0.02	0.11	–0.12*	–0.02	0.04	0.13*	0.21**
7. Cognitive load	4.24 (0.69)	4.27 (0.62)	0.05	0.21*	0.05	0.44**	0.13	0.54**	1	0.16*	–0.02	0.03	–0.14*	–0.11	0.16**	0.13*	0.07
8. Work-family conflict	3.05 (1.09)	2.67 (1.00)	0.33**	0.01	0.08	0.37	0.11	0.22	0.32**	1	–0.15*	–0.27**	–0.27**	–0.11	0.23**	–0.06	–0.09
9. Job autonomy	3.11 (0.92)	3.85 (0.84)	–0.21*	0.09	0.25**	–0.07	–0.31**	0.00	0.09	–0.08	1	0.45**	0.18**	0.02	–0.03	0.26**	0.16**
10. Role clarity	4.10 (0.96)	4.97 (0.88)	–0.32**	0.02	0.23*	–0.00	–0.23**	0.13	0.22**	–0.12	0.38**	1	0.34**	0.29**	–0.03	0.32**	0.29**
11. Supervisors’ support	3.32 (1.56)	4.60 (1.44)	–0.08	–0.06	–0.02	–0.09	–0.07	–0.04	0.08	–0.07	0.27**	0.28**	1	0.33**	–0.05	0.08	0.11
12. Colleagues’ support	4.01 (1.23)	4.89 (1.23)	–0.09	–0.14	–0.03	–0.09	–0.03	–0.16	–0.09	0.02	0.03	–0.02	0.11	1	–0.00	0.04	0.06
13. Family support	3.70 (0.58)	3.41 (0.68)	0.11	0.03	0.01	0.22*	0.25*	0.30**	0.27**	0.34**	–0.20*	0.01	0.01	–0.26**	1	0.15*	–0.04
14. Resilience	3.76 (0.53)	3.96 (0.59)	–0.17	0.09	0.10	0.35**	–0.06	0.03	0.21*	–0.03	0.19	0.27**	0.02	–0.02	0.00	1	0.41**
15. STSE	5.05 (0.99)	5.04 (1.03)	–0.29**	–0.21*	0.19	0.15	0.01	0.10	0.23**	–0.09	0.27**	0.41**	0.12	–0.00	–0.03	0.53**	1

*p < 0.05; **p < 0.01.

Before the results of hierarchical multiple regression, we present the results of the correlations between STS, job demands and job resources in the PO and HCP groups. After hierarchical multiple regression, we present the results of the differences between the PO and HCP groups.

For POs, correlation analyses (Table 1) show that among job demands, STS has a positive correlation with work-family conflict and emotional dissonance. Among the job resources, STS has a negative correlation with role clarity, STSE and job autonomy. For HCPs, STS has a positive correlation with job demands, including work-family conflict, workload, and cognitive load. Among the job resources, STS has a negative correlation with role clarity, resilience, family support, STSE, supervisors’ support, and job autonomy.

Hierarchical multiple regression analyses were used to identify which demands and resources are related to STS (see Table 2). Only the variables that are correlated with STS were included in the regression models. We introduced the job demands in the first model and the job resources in the second model, in line with the *JD-R model* (Demerouti et al., 2001; Bakker and Demerouti, 2014). According to the *JD-R model*, job demands should increase malaise (e.g., STS) and job resources should decrease malaise, as they are protective factors of well-being at work. Regarding POs, in model 1 (13% explained variance), STS is significantly and positively associated only with work-family conflict (β = 0.32). In model 2 (with the addition of job resources), work-family conflict is the only variable positively and significantly associated with STS (β = 0.28) (25% explained variance). Concerning HCPs, in model 1 (17% explained variance), STS is positively and significantly associated with work-family conflict (β = 0.35) and workload (β = 0.16). In model 2 (with the addition of job resources), STS is significantly and positively associated with work-family conflict (β = 0.28) and workload (β = 0.13) and negatively associated with resilience (β = −0.21) and role clarity (β = −0.15) (25% explained variance). The multicollinearity test was performed for both models: the variance inflation factor (VIF) was always less than 10, highlighting the absence of multicollinearity. In both models, the increase in *R*^2^ was significant.

**TABLE 2:** Hierarchical multiple regression – POs, HCPs.

STS				
	POs		HCPs	
	β	p	β	p
**Model 1**				
Job demands				
Workload	–	–	0.16	0.02
Cognitive load	–	–	0.01	0.82
Emotive dissonance	0.16	0.09	–	–
Work-family conflict	0.32	0.00	0.35	0.00
*R*^2^	0.13*		0.17*	
**Model 2**				
Job demands and job resources				
Workload	–	–	0.13	0.04
Cognitive load	–	–	0.06	0.34
Emotive dissonance	0.09	0.37	–	–
Work-family conflict	0.28	0.00	0.25	0.00
Job autonomy	−0.08	0.42	0.02	0.27
Role clarity	−0.17	0.08	−0.14	0.03
Supervisors’ support	–	–	0.04	0.56
Family support	–	–	0.10	0.07
Resilience	–	–	−0.21	0.00
STSE	−0.20	0.05	0.01	0.83
*R*^2^*	0.25*		0.25*	

*p < 0.01.

The *t*-test results (Table 3) show differences between the POs and the HCPs. STS, negative emotions and burnout are higher in POs than in HCPs, whereas positive emotions and job satisfaction are higher in HCPs than in POs. Among job demand outcomes, workload, and work-family conflict are higher in POs than in HCPs. However, job resource outcomes are higher in HCPs than in POs, specifically, job autonomy, supervisor and colleague support, role clarity, and resilience. In contrast, family support is higher in POs than in HCPs. Regarding the *t*-test’ s effect sizes, we note small (=0.20) and medium (=0.50) effect size.

**TABLE 3:** *T*-test for independent samples (comparison between POs and HCPs).

	Groups	*M*	*SD*	*t* (*df*)	Cohen’s *d*	Effect-size *r*
STS	POs (N = 112)	2.17	0.63	7.97* (175.04)	1.20	0.52
	HCPs (*N* = 284)	1.63	0.52			
Negative emotions	POs (*N* = 112)	2.61	0.96	3.22* (393)	0.32	0.16
	HCPs (*N* = 283)	2.29	0.87			
Burnout	POs (*N* = 110)	2.95	0.75	4.60* (389)	0.47	0.23
	HCPs (*N* = 281)	2.59	0.67			
Positive emotions	POs (*N* = 111)	3.44	0.90	−8.86* (393)	0.89	0.40
	HCPs (*N* = 285)	4.27	0.81			
Job satisfaction	POs (*N* = 112)	2.70	0.67	−11.96* (395)	1.20	0.51
	HCPs (*N* = 285)	3.72	0.79			
Workload	POs (*N* = 112)	4.16	0.95	5.11* (393)	0.51	0.25
	HCPs (*N* = 283)	3.65	0.86			
Work-family conflict	POs (*N* = 106)	3.05	1.09	3.23* (383)	0.33	0.16
	HCPs (*N* = 276)	2.67	1.00			
Job autonomy	POs (*N* = 112)	3.11	0.92	−7.62* (393)	0.77	0.36
	HCPs (*N* = 283)	3.85	0.84			
Supervisor support	POs (*N* = 105)	3.32	1.56	−6.33* (387)	0.64	0.30
	HCPs (*N* = 273)	4.60	1.45			
Colleague support	POs (*N* = 107)	4.01	1.24	−7.60* (385)	0.77	0.36
	HCPs (*N* = 275)	4.89	1.23			
Role clarity	POs (*N* = 107)	4.10	0.96	−8.43* (383)	0.86	0.39
	HCPs (*N* = 278)	4.98	0.88			
Resilience	POs (*N* = 106)	3.77	0.53	−2.93* (383)	0.30	0.15
	HCPs (*N* = 279)	3.96	0.59			
Family support	POs (*N* = 105)	3.70	0.58	3.87* (382)	0.40	0.19
	HCPs (*N* = 279)	3.41	0.68			

*p < 0.01. The df for the STS refers to “Equal variances not assume,” because the F value has p < 0.05.

## Discussion

The aim of this work was to analyze the STS of POs working in a municipal police force in Italy. The results showed that some POs suffer more than HCPs regarding certain consequences of STS, such as negative emotions and burnout, consistent with previous investigations (Saakvitne et al., 1996; Leonard and Alison, 1999; Vrklevski and Franklin, 2008; Perez et al., 2010). In addition, POs suffer more than HCPs regarding workload and work-family conflict. In line with the *JD-R model*, job demands have a positive correlation with STS, while job resources have a negative correlation with STS in both groups. However, we highlight that not all variables were significantly correlated with the dependent variable (STS). Only the variables that were correlated with STS were included in the regression models. A hierarchical multiple regression analysis showed that in POs, only work-family conflict resulted associated with STS, thus partially confirming hypothesis 1. In HCPs, regression analysis showed more multiple significant predictors, such that higher STS is associated with higher job demands: work-family conflict and, to a lesser extent, to workload. Regarding job resources in POs, no variables are related to STS, while in HCPs, STS resulted associated with two job resources, and, in particular, STS seems to be decreased by role clarity and resilience. Therefore, also hypothesis 2 is partially confirmed. Moreover, consistent with the literature (Cheung and McNeil Boutté-Queen, 2000; Perez et al., 2010; Bachynski et al., 2012), the findings showed that POs suffer from STS and its consequences more than HCPs employed as first responders and with whom they share the role of intervention in traumatic events. Thus, hypothesis 3 was confirmed.

It seems that POs have difficulties clarifying what the organizations expect from them, their degree of autonomy and the efficacy of their intervention. As a consequence, the risk for this population is related to failing to find strategies for coping with the emotional responses that occur as a result of helping others deal with a traumatic experience (Kop et al., 1999). In fact, burnout syndrome, a possible consequence of STS, in our investigation was higher in POs than in HCPs. These data merit further consideration. Traditionally, burnout has been well-described in HCPs, while in POs, the results from previous investigations are discordant (Martinussen et al., 2007; Sherwood et al., 2019). Thus, a better understanding of this syndrome and its relation to STS in police officers is needed. It could be useful, for example, to adopt a person-centered approach to compare the relationship of job demands, job resources and burnout across time in POs and HCPs experiencing the same traumatic event. Another interesting finding is about the years of service of POs. From the literature, it emerged that in this population more years of service are related to less traumatic stress due to having a better use of coping strategies, a better position within the organization, and a more efficient way to mitigate cognitive and emotive dissonance (Violanti, 2006; Waters and Ussery, 2007; Leino T.M. et al., 2011). The results of this investigation showed that years of services were not related to STS in either POs or HCPs. Further research must investigate these data in relation to roles within the organization. In fact, it is possible that the participants had not yet reached a better position within the organization despite having spent a long time in it or that they had become disillusioned and, as a consequence, were at a greater risk of suffering from chronic distress than from STS (van der Velden et al., 2013). Regarding work-family conflict, in POs, this is the unique variable that explains STS, while in HCPs, more work-family conflict and workload and lower resilience and role clarity were associated with higher STS. Setti et al. (2016) examined emergency workers and found that STS could lead to negative spillover effects on work-family balance. Work-family conflict, which originates from an incompatibility between the job and the family demands, can contribute to work malaise. On the other hand, a family could be a form of social support that helps a PO to cope with STS and may also protect against negative health effects, particularly when the PO is not supported by their colleagues or superiors. At the same time, if the members of the family ask the PO to keep them separate from the negative emotions related to the traumatic events experienced at work, the PO could not express feelings of anger, fear or anguish, thereby increasing the feeling of helplessness and isolation (Papazoglou et al., 2019).

There were, of course, limitations to this study. First, since the sample was non-randomly selected, the results should be taken with caution and should not be generalized. Moreover, as described above, the respondent’s role within the organization was not considered. Studies that have directly assessed the role within the organization in this population have found that this aspect could be a protective factor against STS. For example, Sheard et al. (2019) found that STS is experienced more by POs who are on the frontline of crime initiatives than by POs who deal with investigations into serious crimes. Future research should investigate STS more in depth in relation to POs’ roles within the organization. Another limitation of this study was in reference to the source of the STS experienced. The type of intervention and the exposure to persons who have experienced one (or more) traumatic events could vary and thus influence the STS symptoms. As suggested by MacEachern et al. (2019), disturbing material (such as child pornography or intimate partner violence; Zara and Gino, 2018) greatly increases the symptoms of STS. Further research must investigate the exposure to and the type of traumatic events in which POs have to intervene. Concerning gender, Agocs et al. (2015) found that as a consequence of their exposure to traumatic events, female POs are more likely to engage in strategies to prevent their own children from becoming victims or offenders of various crimes. Further research could investigate whether PO mothers and fathers suffering from STS adopt different behaviors in regard to their children.

Despite these limitations, we hope that this study offers implications and interesting insights for both POs and the organizations in which they work. First, it is important to promote organizational support by superiors and colleagues. This strategy could increase POs’ opportunities to express negative feelings, to find new coping strategies to deal with stressful events and to mitigate the negative impact of traumatic incident stress. Moreover, the aim is to consider POs not only as providers of care in traumatic events but also as potential victims of STS. For this particular population, due to the content of their work, it could be more difficult to recognize STS symptoms, as POs often use denial or avoidance strategies to cope with them. These strategies could be perceived as immediately effective because they facilitate the removal of the source of stress. At the same time, denial as a method of coping is associated with an increased use of behavioral disengagement (Brooks et al., 2019).

Finally, PO organizations should contribute to preventing the phenomenon of STS, both from an individual and organizational perspective: Svetlitzky et al. (2019) underlined that from an individual perspective, the intervention to decrease stress reaction must be performed during traumatic events, for example, asking POs to focus their attention on the action. Papazoglou and Andersen (2014) proposed programs that implement resilience training and the use of adaptive coping strategies. Previously, Arnetz et al. (2009) designed a protocol to reduce the negative effect of work-related stress. The aim is to improve POs’ feelings of control in stressful situations by providing a list of strategies that can be used to manage traumatic incidents. Anshel et al. (2013) proposed including physical fitness activities such as a “time-out” that permits POs to stop the source of their anxiety and to increase their energy and, as a consequence, to increase the strategies used to cope with the traumatic event. Moreover, to improve coping strategies and resilience, a meta-analysis by Purba and Demou (2019) suggested that it is important to increase the support given by superiors and colleagues because the lack of such support is related to mental health outcomes. Furthermore, prevention programs could include the following: information courses on STS, its symptoms and consequences (this happens in HCP training); a training program focused on enhancing POs’ strategies for using adaptative coping strategies and building resilience; creating an organizational climate that helps POs who suffer from STS; and mitigating the negative stigma associated with POs who seek counseling and psychological help (Turgoose et al., 2017). Moreover, as suggested by Salston and Figley (2003), it is vital to stress the importance of supervision or consultation in workers with painful incidents, trauma victims and/or survivors. These techniques offer the opportunity to process both the traumatic event and the personal emotions and cognitions regarding it, thus helping the management of the work-family conflict related to STS.

Future research should use a larger sample to replicate the analyses herein to test the impacts of STS and its consequences considering the POs’ roles within the organization, their exposure to traumatic events and their gender. This research could help to better understand STS in the PO population by providing guidance to organizations regarding the adoption of prevention programs.

## Data Availability Statement

The datasets generated for this study are available on request to the corresponding author.

## Ethics Statement

Ethical review and approval was not required for the study on human participants in accordance with the local legislation and institutional requirements. The patients/participants provided their written informed consent to participate in this study.

## Author Contributions

DA, MZ, and LC: conceptualization, investigation, and writing (review and editing). LC and MZ: methodology. LC: data analysis. DA and LC: writing (original draft preparation). All authors contributed to the article and approved the submitted version.

## Conflict of Interest

The authors declare that the research was conducted in the absence of any commercial or financial relationships that could be construed as a potential conflict of interest.

## Footnotes

1 http://www.comune.torino.it/vigiliurbani/poliziamunicipale/struttura/comando/formazione.shtml
